# Effects of statins on glycemic control and cardiovascular events in patients with new-onset type 2 diabetes: a retrospective cohort study

**DOI:** 10.3389/fendo.2026.1921249

**Published:** 2026-08-28

**Authors:** Ying Ran, Lili Jiang, Jing Ran, Yu Zhu, Lupin Pan, Bayi Yang, Xue Ran, Hejun Ding, Shaofa Wu, Xia Ling

**Affiliations:** 1 Department of Nephrology, Youyang Hospital, a Branch of The First Affiliated Hospital of Chongqing Medical University, Chongqing, China; 2 Department of Cardiology, Youyang Hospital, a Branch of The First Affiliated Hospital of Chongqing Medical University, Chongqing, China

**Keywords:** glycemic control, major adverse cardiovascular events, new-onset type 2 diabetes, retrospective cohort study, statins

## Abstract

**Background:**

Cardiovascular disease is the leading cause of morbidity and mortality in patients with type 2 diabetes (T2D). While statin therapy is universally recommended for cardiovascular disease prevention, its potential adverse effects on glycemic progression remain controversial, particularly in patients with newly diagnosed disease. This study aimed to evaluate the dual effects of statin initiation on glycemic control and major adverse cardiovascular events (MACEs) in a retrospective cohort of patients with new-onset T2D.

**Methods:**

We conducted a retrospective cohort study in which patients who were newly diagnosed with T2D between January 2018 and December 2020 were screened. The primary outcome was the change in the glycated hemoglobin (HbA1c) levels. Secondary outcomes included fasting plasma glucose (FPG) levels, 2-hour post-prandial glucose (2hPG) levels, diabetes therapy intensification, insulin initiation, hyperglycemic crisis, and MACEs. Longitudinal glycemic trajectories were assessed using two-way repeated-measures ANOVA. The MACE risk was evaluated with a Kaplan–Meier analysis and multivariable Cox regression model.

**Results:**

A total of 604 eligible patients were classified into a statin group (n = 458) and a nonstatin group (n = 146). At the 6-month follow-up, both groups exhibited improved glycemic indices, but this improvement was significantly attenuated in the statin group. Similar attenuated improvements were observed in the FPG and 2hPG levels at 6 months. Group × time interaction effects were significant for FPG, 2hPG, and HbA1c levels at 6 months. By 60 months, between-group differences in HbA1c levels were no longer significant. During the median (interquartile range) follow-up of 73 (68–80) months, statin therapy was associated with higher frequencies of diabetes therapy intensification (33.3% vs. 22.6%, p = 0.016), insulin initiation (21.5% vs. 12.3%, p = 0.015), and hyperglycemic crisis (8.5% vs. 3.4%, p = 0.039). Despite these metabolic disadvantages, statin therapy was independently associated with a lower MACE risk according to the multivariable Cox regression analysis (HR 0.41, 95% CI 0.21–0.83; p = 0.013).

**Conclusions:**

In patients with new-onset T2D, early statin initiation is associated with worse short-term glycemic improvement and a greater need for escalating glucose-lowering therapy; however, it is also associated with a substantial reduction in the long-term MACE risk.

## Introduction

Atherosclerotic cardiovascular disease (ASCVD) is a leading cause of death worldwide, and low-density lipoprotein cholesterol (LDL-C) is a major causal risk factor (1). Furthermore, type 2 diabetes mellitus (T2D) is an established independent driver of ASCVD (2). Statins reduce ASCVD events in people with and without diabetes and are therefore recommended to prevent cardiovascular disease in patients with T2D (3–5).

Statins exert their lipid-lowering effects primarily by inhibiting 3-hydroxy-3-methylglutaryl-coenzyme A (HMG-CoA) reductase, thereby reducing hepatic mevalonate production, a key step in the hepatic cholesterol synthesis pathway. The resulting depletion of intracellular cholesterol upregulates the expression of low-density lipoprotein (LDL) receptors on the hepatocellular membrane, facilitating the systemic clearance of circulating chylomicrons, very-low-density lipoproteins (VLDLs), and LDL. Previous studies have shown that statins reduce the incidence of coronary heart disease (CHD) and overall mortality in patients with diabetes (5–7). Despite these cardiovascular benefits, large-scale randomized controlled trials have demonstrated a significant association between statin administration and an increased incidence of new-onset diabetes (8). In pooled data from 13 trials, statin therapy was associated with a 9% increased risk of diabetes that was attributable to decreases in insulin sensitivity and insulin secretion (9–13). While most evidence has focused on patients with new-onset diabetes, less is known about whether initiating statins at the time of the new T2D diagnosis influences glycemic trajectories and long-term cardiovascular outcomes.

This retrospective cohort study aimed to evaluate the effects of early statin initiation on glycemic control and major adverse cardiovascular events (MACEs) in a real-world cohort of patients with new-onset T2D.

## Methods

This retrospective cohort study was conducted at Youyang Hospital, the First Affiliated Hospital of Chongqing Medical University, between January 2018 and December 2020. Electronic medical records databases were systematically reviewed to identify patients aged ≥ 18 years with an initial diagnosis of T2D. New-onset type T2D was defined as the first documented clinical diagnosis of T2D in the electronic medical record, with no prior diagnosis of any form of diabetes, no previous prescription of glucose-lowering medications, and no abnormal glycemic laboratory results recorded in medical records before the index visit. The index date was defined as the date of the first clinical encounter where T2D was diagnosed and baseline laboratory tests were completed (14, 15). The diagnoses were based on the following standard American Diabetes Association criteria: a baseline fasting plasma glucose (FPG) level ≥ 7.0 mmol/L, 2-hour post-prandial glucose (2hPG) level ≥11.1 mmol/L, or a glycated hemoglobin (HbA1c) level ≥ 6.5% (16). Enrolled patients were followed for 60 months to assess metabolic outcomes, while survival and clinical cardiovascular events were monitored through continuous outpatient records and telephone follow-ups beyond the 60-month evaluation period.

The following exclusion criteria were applied: (1) systemic steroid use or polycystic ovary syndrome (which impairs insulin sensitivity and increases plasma glucose levels); (2) pregnancy or HIV infection (16); (3) the presence of advanced liver disease (Child–Pugh class B or C), severe renal impairment (eGFR < 30 mL/min/1.73 m²), or malignant neoplasms (17, 18); (4) the presence of type 1 or gestational diabetes or other forms of diabetes; and (5) incomplete or missing critical clinical data, including patients who were lost to follow-up or lacked complete glycemic measurements during the 60-month evaluation period.

This study complied with the Declaration of Helsinki and was approved by the Ethics Committee of Youyang Hospital, the First Affiliated Hospital of Chongqing Medical University (Approval No. 2026011YYXRMYY) (19). Due to the retrospective design of this research, the ethics committee waived the requirement for informed consent.

### Data collection and clinical variables

Baseline demographic and clinical data were extracted from the patients’ electronic medical records. The collected variables included age, sex, body mass index (BMI), systolic blood pressure (SBP), diastolic blood pressure (DBP), and essential hematological parameters, including hemoglobin (HGB). Height and weight were objectively measured by trained nursing staff using calibrated clinical scales at the baseline visit and each subsequent visit, and BMI was calculated as weight in kilograms divided by the square of height in meters. Complete lipid panels, including total cholesterol (TC), triglyceride (TG), high-density lipoprotein cholesterol (HDL-C), and low-density lipoprotein cholesterol (LDL-C) levels, were obtained at baseline. Smoking history, alcohol consumption, family history of diabetes, and comorbidities were also recorded. Urea, serum creatinine, alanine aminotransferase (ALT), aspartate aminotransferase (AST), gamma-glutamyl transferase (GGT), albumin (ALB), fasting plasma glucose (FPG), 2-hour post-prandial glucose (2hPG), and HbA1c levels were measured at baseline and during follow-up.

Data on new drug prescriptions at the first visit, including statin and concurrent glucose-lowering agents, were extracted from the clinical records. In the routine clinical management of T2D, follow-up visits were regularly performed for all patients at least every six months after enrollment; at each visit, the same measurements described above were repeated for all patients. At the first visit, all patients received standardized verbal counseling and written educational pamphlets regarding a diabetic diet and regular physical exercise provided by attending physicians and specialized diabetes nurses in accordance with clinical guidelines. Formal dietitian-led individualized nutritional programs were not routinely available during the study period. Data on statin prescriptions were also retrieved from clinical records during the same observation period. Patients who initiated statin therapy after the first visit were excluded from the analysis.

### Follow-up, glycemic endpoints, and cardiovascular outcomes

The primary endpoints were changes in HbA1c levels from baseline to 6, 12, 24, 36 and 60 months.

The secondary endpoints included changes in FPG and 2hPG levels, diabetes therapy intensification, insulin initiation, hyperglycemic crisis, and MACEs. Diabetes therapy intensification was defined as any upward adjustment of antihyperglycemic regimens during follow-up, including the addition of new oral glucose-lowering drug classes, the initiation of insulin among oral drug-only patients, or a dose escalation of existing antihyperglycemic agents to the maximum tolerated doses (20). A hyperglycemic crisis includes diabetic ketoacidosis (DKA) and a hyperglycemic hyperosmolar state (HHS) (21). DKA was defined as a random plasma glucose concentration ≥11.1 mmol/L, an arterial pH <7.30, a serum bicarbonate concentration <18 mmol/L, and an elevated concentration of ketones (a serum β-hydroxybutyrate concentration ≥3.0 mmol/L or ≥2+ urine ketones) (22). HHS was defined as a serum effective osmolality ≥320 mOsm/kg, a random glucose concentration ≥33.3 mmol/L, and the absence of or presence of minimal ketones with hyperosmolar diabetes admission codes (22). MACEs were defined as a composite endpoint comprising nonfatal myocardial infarction, ischemic or hemorrhagic stroke, and all-cause mortality (4). The survival time and MACE-free status were monitored via continuous outpatient records and telephone follow-ups extending beyond the initial 60-month metabolic evaluation period.

### Statistical analysis

Continuous variables were evaluated for normality using the Kolmogorov–Smirnov test. Normally distributed continuous parameters are presented as the means ± standard deviations (SDs), and comparisons between the statin and nonstatin cohorts were performed using independent Student’s t tests. Nonnormally distributed continuous variables are presented as the medians and interquartile ranges (IQRs). Conversely, the Wilcoxon signed-rank test for paired samples was utilized when the differences did not adhere to a normal distribution. Categorical variables are presented as frequencies and percentages (%), and significant differences were analyzed using the Pearson chi-square test or Fisher’s exact test, where appropriate. Two-way repeated-measures analysis of variance (ANOVA) was performed to evaluate the dynamic trajectories of FPG, 2hPG, and HbA1c levels across multiple time points (baseline, 6 months, and 60 months) and between the two treatment groups. The main effects of the group, time, and their interaction (group × time) are reported with the corresponding F values and p values. Long-term MACE-free survival curves were constructed using the Kaplan–Meier method, and differences between survival distributions were assessed using the log-rank test. Variables were selected for the multivariable model based on clinical relevance and significant baseline differences observed between the groups. As this retrospective cohort study was based on clinical records, no formal *a priori* sample size calculation was performed; rather, a convenience sample comprising all eligible patients who met the inclusion criteria during the study period was included. All the statistical analyses were performed using SPSS version 25.0 (IBM SPSS Statistics, Armonk, NY, USA) and GraphPad Prism 9.0. A two-tailed p value < 0.05 was considered to indicate statistical significance.

## Results

### Baseline demographic and clinical characteristics

**Figure 1:**
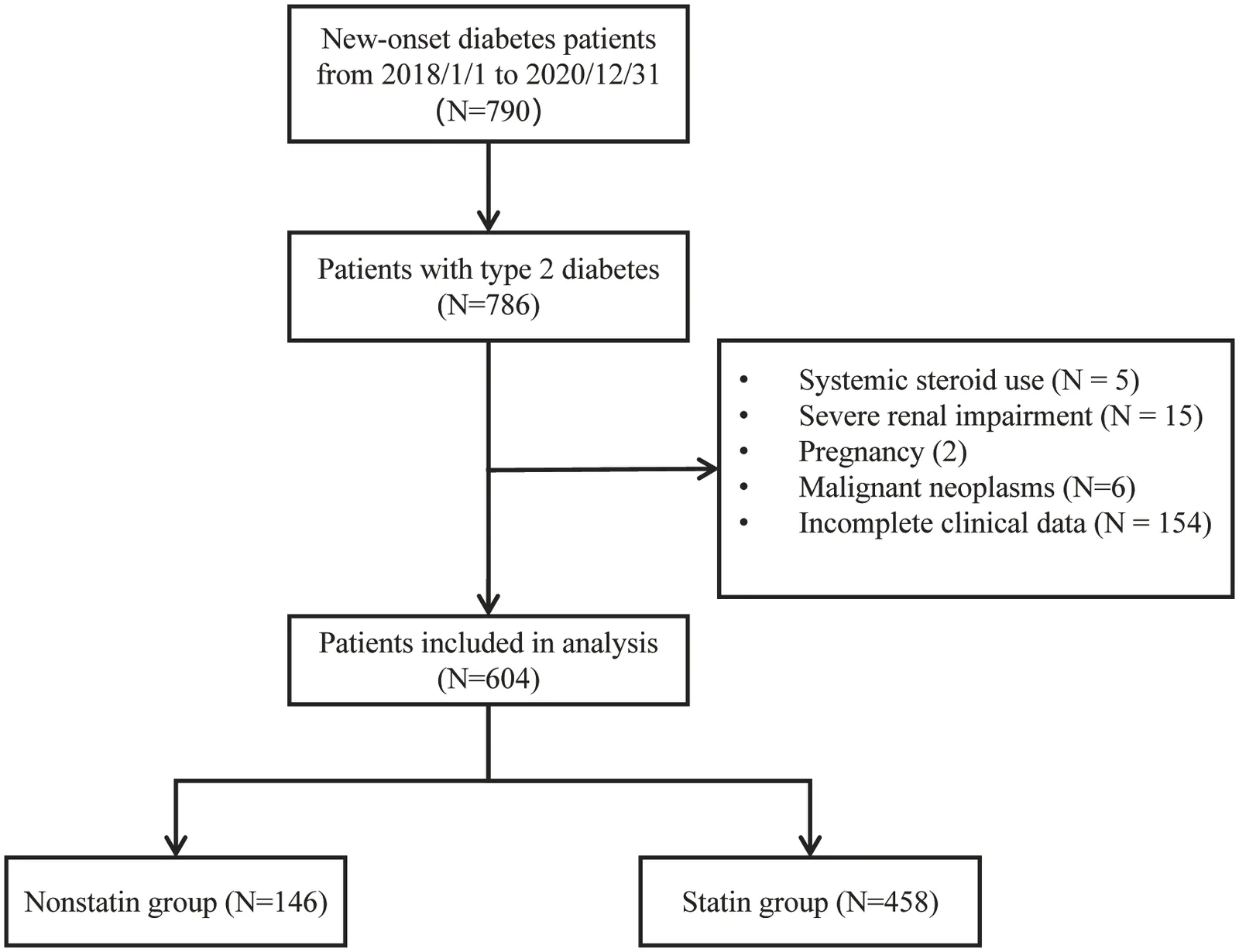
Flow diagram for patient inclusion and exclusion in this study.

A total of 790 patients with new-onset diabetes who were treated at our hospital were initially screened from January 2018 to December 2020, among whom 786 patients were identified as having T2D. In accordance with the inclusion and exclusion criteria, 182 patients were excluded from this study, including 5 with systemic steroid use, 15 with a severe renal impairment, 2 who were pregnant, 6 with malignant neoplasms, and 154 with incomplete clinical data. Ultimately, 604 patients were included in the final analysis, including 146 patients in the nonstatin group and 458 patients in the statin group (**Figure 1**). Because complete longitudinal data were needed for inclusion, all 604 patients in the final cohort successfully completed the full 60-month follow-up of metabolic endpoints. Patients who did not complete the follow-up were already accounted for within the 154 patients who were excluded for incomplete clinical data during the screening phase.

The demographic and clinical characteristics of the 604 patients with new-onset T2D are presented in **Table 1**. The average age was 61.93 ± 13.35 years; 312 patients were male (51.7%), and 231 had a history of smoking (38.2%). No statistically significant differences in age, sex distribution, smoking history, alcohol consumption, or body mass index (BMI) were observed between statin users and nonusers. Moreover, no significant differences were observed in baseline comorbidities, such as hypertension, chronic kidney disease (CKD), coronary artery disease (CAD), or cerebrovascular disease (CVD). Diastolic blood pressure was similar in both groups (79.82 ± 11.44 vs. 81.61 ± 11.21 mmHg, p = 0.109), but patients in the statin group had a significantly higher baseline systolic blood pressure (129.88 ± 18.16 vs. 124.92 ± 15.96 mmHg, p = 0.003).

**Table 1:** Baseline demographic, clinical and laboratory characteristics of patients with new-onset type 2 diabetes stratified by statin use.

Parameter and group	Overall (n = 604)	Statin nonusers (n = 146)	Statin users (n = 458)	P value
Age, y	61.93 ± 13.35	62.74 ± 14.34	61.67 ± 13.03	0.422
Males, n (%)	312 (51.7)	75 (51.4)	237 (51.7)	0.937
Smoking history, n (%)	231 (38.2)	58 (39.7)	173 (37.8)	0.672
Alcohol consumption, n (%)	138 (22.8)	37 (25.3)	101 (22.1)	0.410
CKD, n (%)	122 (20.2)	34 (23.3)	88 (19.2)	0.286
CAD, n (%)	116 (19.2)	22 (15.1)	94 (20.5)	0.145
Family history of DM, n (%)	32 (5.3)	10 (6.8)	22 (4.8)	0.337
Hypertension, n (%)	274 (45.4)	60 (41.1)	214 (46.7)	0.234
CVD, n (%)	24 (4.0)	4 (2.7)	20 (4.4)	0.381
SBP, mmHg	128.68 ± 17.77	124.92 ± 15.96	129.88 ± 18.16	0.003
DBP, mmHg	81.18 ± 11.28	79.82 ± 11.44	81.61 ± 11.21	0.109
BMI	23.67 ± 2.93	23.88 ± 2.79	23.60 ± 2.97	0.311
HGB, g/L	130.55 ± 22.57	126.32 ± 25.23	131.82 ± 21.55	0.011
Urea, mmol/L	5.68 (4.45, 7.15)	5.41 (4.28, 6.81)	5.73 (4.49, 7.23)	0.245
SCr, μmol/L	70.00 (60.90, 86.70)	70.00 (58.10, 90.20)	70.00 (61.00, 86.08)	0.905
TC mmol/L	4.48 ± 1.30	4.40 ± 1.36	4.51 ± 1.28	0.380
TG, mmol/L	2.94 ± 1.68	2.66 ± 1.79	3.03 ± 1.64	0.018
HDL-C, mmol/L	1.03 ± 0.36	0.99 ± 0.34	1.05 ± 0.36	0.066
LDL-C, mmol/L	2.84 ± 0.94	2.50 ± 0.86	2.94 ± 0.94	<0.001
ALT, u/L	18.00 (13.00, 27.00)	16.70 (12.00, 27.00)	19.00 (13.00, 27.00)	0.210
AST, u/L	22.00 (15.00, 48.25)	22.00 (15.00, 40.00)	22.00 (15.00, 50.00)	0.461
GGT, u/L	24.00 (16.00, 42.25)	24.00 (15.00, 40.00)	25.00 (16.00, 50.00)	0.744
ALB, g/L	40.06 ± 7.49	40.55 ± 6.84	39.91 ± 7.68	0.368
FPG, mmol/L	7.15 ± 1.62	7.16 ± 1.55	7.15 ± 1.65	0.931
2hPG, mmol/L	11.39 ± 2.27	10.39 ± 2.62	11.70 ± 2.04	<0.001
HbA1c, %	6.92 ± 0.56	6.87 ± 0.64	6.94 ± 0.53	0.074
Metformin, n (%)	364 (60.3)	89 (61.0)	275 (60.0)	0.844
SGLT2 inhibitor, n (%)	319 (52.8)	86 (58.9)	233 (50.9)	0.091
DPP4i, n (%)	60 (9.9)	11 (7.5)	49 (10.7)	0.266
GLP-1RA, n (%)	32 (5.3)	11 (7.6)	21 (4.6)	0.154
Others OAD, n (%)	178 (29.5)	37 (25.3)	141 (30.8)	0.209

The values are presented as the means (standard deviations), numbers (percentages of the total numbers of participants in the column) or medians (interquartile ranges). CKD, chronic kidney disease; CAD, coronary artery disease; SBP, systolic blood pressure; DBP, diastolic blood pressure; BMI, body mass index; HGB, hemoglobin; SCr, serum creatinine; TC, total cholesterol; TG, triglyceride; HDL-C, high-density lipoprotein cholesterol; LDL-C, low-density lipoprotein cholesterol; ALT, alanine aminotransferase; AST, aspartate aminotransferase; GGT, gamma-glutamyl transferase; ALB, albumin; DM, diabetes mellitus; CVD, cerebrovascular disease; FPG, fasting plasma glucose; 2hPG, 2-hour post-prandial glucose; HbA1c, glycated hemoglobin A1c; SGLT2, sodium-glucose cotransporter 2; DPP4i, dipeptidyl-peptidase 4 inhibitor; GLP-1RA, glucagon-like peptide-1 receptor agonist; OAD, oral antidiabetic drug.

An analysis of the laboratory parameters revealed that both groups had comparable renal and hepatic functions, with no significant differences in the median urea, serum creatinine, alanine aminotransferase (ALT), aspartate aminotransferase (AST), gamma-glutamyl transferase (GGT), or mean albumin (ALB) levels. Hemoglobin levels were slightly higher in the statin group (131.82 ± 21.55 g/L vs. 126.32 ± 25.23 g/L, p = 0.011). In the lipid profile, total cholesterol and HDL-C levels were similar, whereas triglyceride (3.03 ± 1.64 vs. 2.66 ± 1.79 mmol/L, p = 0.018) and LDL-C (2.94 ± 0.94 vs. 2.50 ± 0.86, p < 0.001) levels were significantly higher in the statin group, reflecting the expected clinical indication for statin prescription. Importantly, the baseline glycemic parameters were similar between the groups, with no significant differences in FPG levels (7.16 ± 1.55 mmol/L vs. 7.15 ± 1.65 mmol/L, p=0.931), whereas baseline 2hPG levels were significantly higher in the statin group (11.70 ± 2.04 vs. 10.39 ± 2.62 mmol/L, p < 0.001). The baseline HbA1c levels did not differ significantly between the groups (6.94 ± 0.53 vs. 6.87 ± 0.64%, p = 0.074).

With respect to baseline antihyperglycemic prescriptions at the first visit, no statistically significant differences were observed between the statin and nonstatin groups. Metformin was the most frequently prescribed agent in both the statin and nonstatin cohorts (60.0% vs. 61.0%, p = 0.844), followed by SGLT2 inhibitors (50.9% vs. 58.9%, p = 0.091). Similarly, the prescription rates for DPP4 inhibitors (10.7% vs. 7.5%, p = 0.266), GLP-1 receptor agonists (4.6% vs. 7.6%, p = 0.154), and other oral antidiabetic drugs (30.8% vs. 25.3%, p = 0.209) were comparable between the two groups.

### Effects of statin therapy on glycemic control

**Figure 2:**
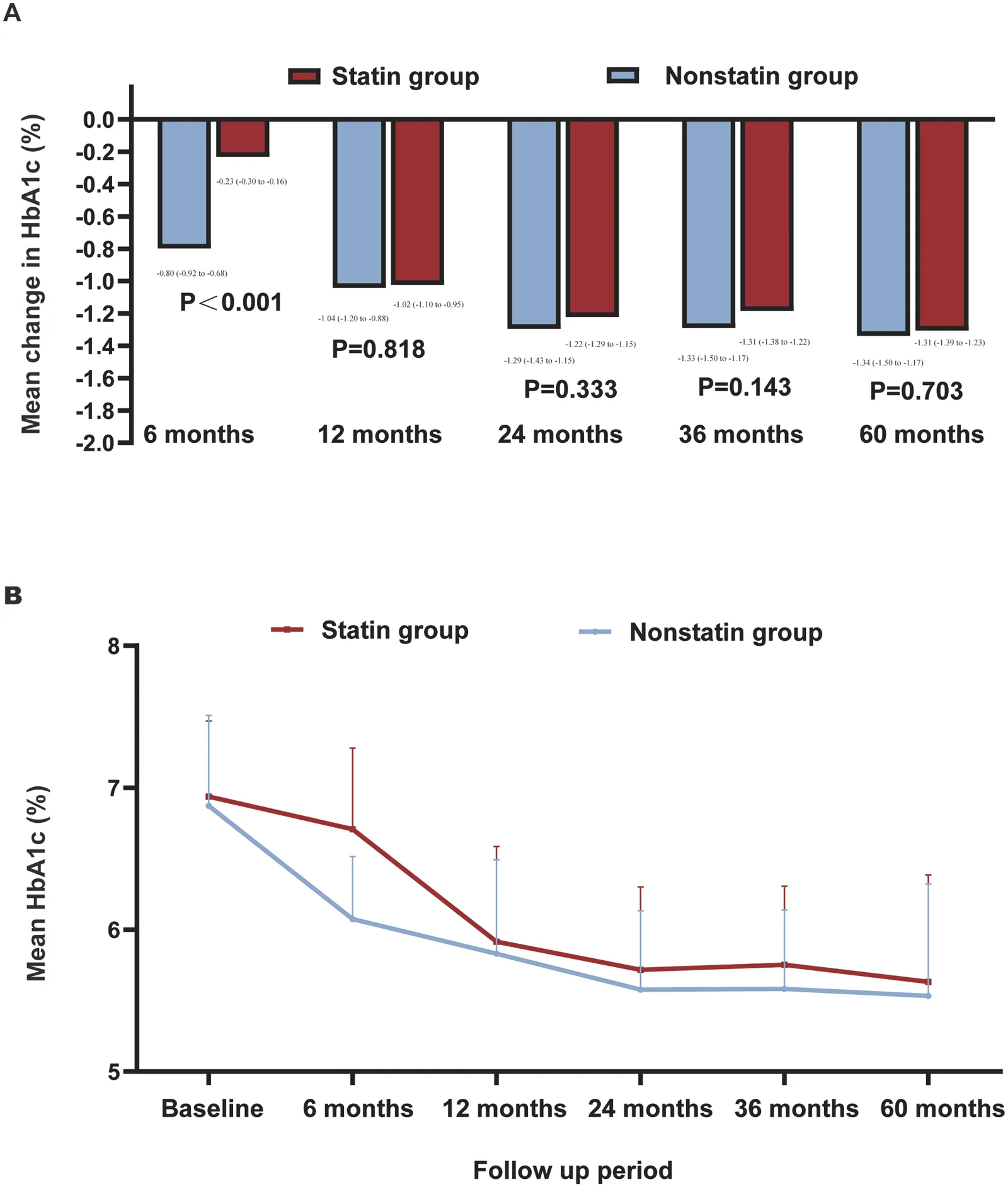
Mean differences in changes in the HbA1c levels between the treatment groups **(a)** and the mean HbA1c levels in both treatment groups **(b)** (p < 0.05 compared with the baseline levels for the study duration).

We analyzed the changes in glycemic parameters at the 60-month follow-up to evaluate the effect of statins on glucose metabolism. The mean reductions in HbA1c levels and mean HbA1c levels at all the study time points are shown in **Figure 2A** and 2B, respectively,. HbA1c levels decreased significantly in both statin users and nonusers throughout the study (**Figure 2A**), but a smaller reduction in HbA1c levels was observed in statin users (**Figure 2B**). This decrease was significant at 6 months, with mean reductions of -0.23% and -0.80% (p < 0.001) in the statin users and nonusers, respectively; however, a significant difference was not observed at 60 months (p = 0.703).

At 6 months, both groups exhibited improvements in glycemic metrics. However, the mean reductions in FPG and 2hPG levels were smaller in the statin group than in the nonstatin group (**Table 2**). As shown in **Table 3**, two-way repeated-measures ANOVA at the 6-month interval confirmed significant group × time interactions for FPG (F = 18.12, p < 0.001), 2hPG (F = 231.03, p < 0.001), and HbA1c levels (F = 62.97, p < 0.001). These findings statistically confirm that statin exposure significantly modified the short-term longitudinal trajectory of glycemic control.

**Table 2:** Changes in fasting plasma glucose, 2-hour post-prandial glucose and glycated hemoglobin levels from baseline to 6 and 60 months in patients stratified by statin treatment.

Parameter and group	Baseline	Follow-up	ΔMean (95% CI)	*P*
FPG level at 6 months, mmol/L				
Without statins	7.16 ± 1.55	6.62 ± 1.49	-0.54 (-0.83 to -0.25)	<0.001
With statins	7.15 ± 1.65	6.94 ± 2.15	-0.20 (-0.40 to -0.01)	0.041
2hPG level at 6 months, mmol/L				
Without statins	10.39 ± 2.62	9.68 ± 2.29	-0.71 (-1.00 to -0.42)	<0.001
With statins	11.70 ± 2.04	11.29 ± 2.62	-0.42 (-0.56 to -0.28)	<0.001
HbA1c level at 6 months, %				
Without statins	6.87 ± 0.64	6.08 ± 0.44	-0.80 (-0.92 to -0.68)	<0.001
With statins	6.94 ± 0.53	6.70 ± 0.57	-0.23 (-0.30 to -0.16)	<0.001
FPG level at 60 months, mmol/L				
Without statins	7.16 ± 1.55	6.41 ± 0.93	-0.74 (-1.05 to -0.44)	<0.001
With statins	7.15 ± 1.65	6.43 ± 0.85	-0.72 (-0.89 to -0.54)	<0.001
2hPG level at 60 months, mmol/L				
Without statins	10.39 ± 2.62	8.33 ± 1.65	-2.06 (-2.55 to -1.56)	<0.001
With statins	11.70 ± 2.04	9.21 ± 1.57	-2.49 (-2.73 to -2.25)	<0.001
HbA1c level at 60 months, %				
Without statins	6.87 ± 0.64	5.53 ± 0.79	-1.34 (-1.50 to -1.17)	<0.001
With statins	6.94 ± 0.53	5.63 ± 0.75	-1.31 (-1.39 to -1.23)	<0.001

FPG, fasting plasma glucose; 2hPG, 2-hour post-prandial glucose; HbA1c, glycated hemoglobin A1c; CI, confidence interval.

**Table 3:** Results of the two-way ANOVA of glucose (FPG), 2-hour postprandial glucose (2hPG), and HbA1c levels at 6 and 60 months.

Parameter and group	Effect	F value	*P*
FPG level at 6 months, mmol/L			
	Group	8.02	0.005
	Time	1.52	0.218
	Group × time interaction	18.12	<0.001
2hPG level at 6 months, mmol/L			
	Group	133.37	<0.001
	Time	33.51	<0.001
	Group × time interaction	231.03	<0.001
HbA1c level at 6 months, %			
	Group	83.28	<0.001
	Time	207.15	<0.001
	Group × time interaction	62.97	<0.001
FPG level at 60 months, mmol/L			
	Group	0.003	0.957
	Time	66.33	<0.001
	Group × time interaction	0.024	0.876
2hPG level at 60 months, mmol/L			
	Group	73.17	<0.001
	Time	308.11	<0.001
	Group × time interaction	2.81	0.094
HbA1c level at 60 months, %			
	Group	3.11	0.078
	Time	951.89	<0.001
	Group × time interaction	0.145	0.703

ANOVA, analysis of variance; FPG, fasting plasma glucose; 2hPG, 2-hour post-prandial glucose; HbA1c, glycated hemoglobin A1c.

However, at 60 months, the long-term glycemic reductions became comparable. ANOVA at the 60-month period (**Table 3**) revealed strong main effects of time (all p < 0.001), but the effects of the group × time interactions on FPG (F = 0.024, p = 0.876), 2hPG (F = 2.81, p = 0.094), and HbA1c levels (F = 0.145, p = 0.703) were no longer significant, indicating that the initial metabolic disadvantage of statins faded over long-term treatment.

### Diabetes therapy intensification and clinical events

In terms of the blunted short-term glycemic response, statin therapy was associated with a significantly greater clinical need for antihyperglycemic treatment escalation (**Table 4**). During the 73 (68–80) months of follow-up, 185 patients (30.6%) required therapy intensification. This rate was significantly higher in the statin group than in the nonstatin group (152 patients [33.3%] vs. 33 patients [22.6%], p = 0.016). Furthermore, the need to initiate insulin therapy was nearly doubled in the statin cohort, occurring in 98 patients (21.5%) compared with 18 patients (12.3%) in the nonstatin cohort (p = 0.015). The incidence of a hyperglycemic crisis was also higher among statin users (39 patients [8.5%] vs. 5 patients [3.4%], p = 0.039).

**Table 4:** Cumulative incidence of adverse glycemic events and major adverse cardiovascular events across statin users and statin nonusers.

Parameter	Overall (n =604)	Statin nonusers (n =146)	Statin users (n =458)	*P*
Intensification of diabetes therapy, n (%)	185 (30.6)	33 (22.6)	152 (33.3)	0.016
Initiate insulin therapy, n (%)	116 (19.3)	18 (12.3)	98 (21.5)	0.015
Hyperglycemic crisis	44 (7.3)	5 (3.4)	39 (8.5)	0.039
Major adverse cardiovascular events^a^, n (%)	35 (5.8)	14 (9.6)	21 (4.6)	0.024
All-cause mortality, n (%)	4 (0.7)	2 (1.4)	2 (0.4)	0.247
Nonfatal acute myocardial infarction, n (%)	16 (2.6)	6 (4.1)	10 (2.2)	0.207
Nonfatal stroke, n (%)	15 (2.5)	6 (4.1)	9 (2.0)	0.147

^a^ Major adverse cardiovascular events include all-cause mortality, nonfatal acute myocardial infarction (AMI) and nonfatal stroke.

With respect to cardiovascular outcomes (**Table 4**), 35 patients (5.8% overall) experienced a MACE. Despite the early metabolic disadvantages, the incidence of MACEs was significantly lower in the statin group (21 patients, 4.6%) than in the nonstatin group (14 patients, 9.6%) (p = 0.024). The MACEs included all-cause mortality (2 statin users [0.4%] vs. 2 statin nonusers [1.4%]), nonfatal acute myocardial infarction (10 statin users [2.2%] vs. 6 statin nonusers [4.1%]), and nonfatal stroke (9 statin users [2.0%] vs. 6 statin nonusers [4.1%]).

### Multivariable survival analysis of cardiovascular outcomes

**Figure 3:**
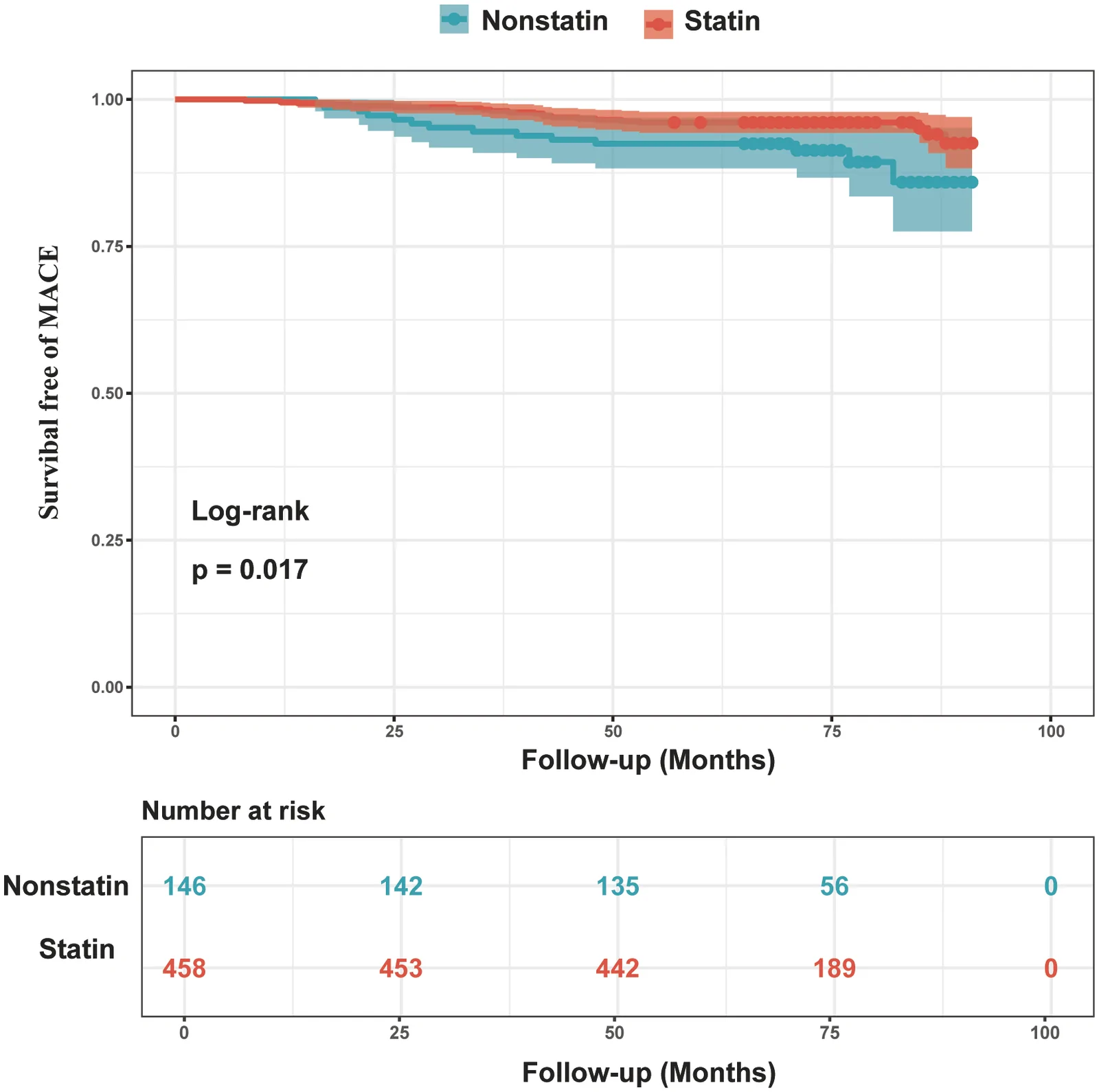
Kaplan–Meier plots of major adverse cardiovascular events (MACEs).

The results of the Kaplan–Meier survival analysis (**Figure 3**) demonstrated that patients who received statins achieved significantly higher MACE-free survival probabilities over the extended follow-up period (log-rank p = 0.017).

**Figure 4:**
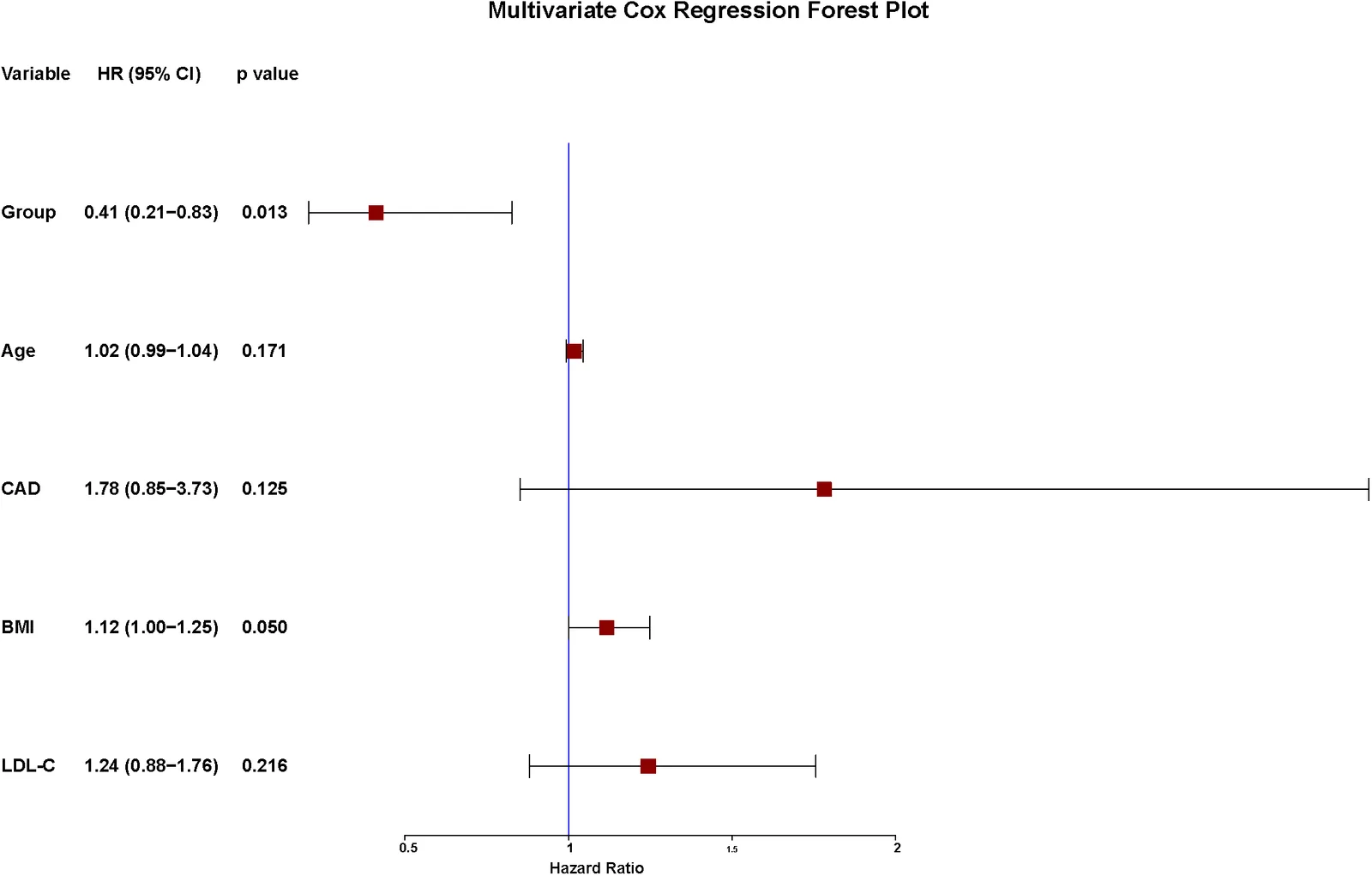
Forest plot of the results of the multivariable Cox regression analysis identifying independent predictors of major adverse cardiovascular events (MACEs). The vertical solid line represents a hazard ratio = 1, the solid squares denote adjusted hazard ratios, and the horizontal lines represent the corresponding 95% confidence intervals.

We constructed a multivariable Cox proportional hazards regression model to adjust for potential confounders (**Figure 4**). The model incorporated five variables: statin therapy, age, BMI, LDL-C level, and CAD status. After adjustment, BMI was an independent risk factor for MACEs (hazard ratio [HR]: 1.12; 95% CI: 1.00 to 1.25; p = 0.050). Age (HR: 1.02; 95% CI: 0.99 to 1.04; p = 0.171), the LDL-C level (HR: 1.24; 95% CI: 0.88 to 1.76; p = 0.216), and CAD status (HR: 1.78; 95% CI: 0.85 to 3.73; p = 0.125) were not statistically significant predictors of MACEs in this specific cohort. Crucially, statin therapy emerged as a strong and independent protective factor against MACEs (HR: 0.41; 95% CI: 0.21 to 0.83; p = 0.013). This result translates to a 59% relative risk reduction in major cardiovascular events among statin users, effectively overriding the clinical risks associated with the observed early blunted glycemic response.

## Discussion

In this retrospective cohort study of 604 patients with newly diagnosed T2D, early statin initiation was associated with an attenuated short-term glycemic improvement and more frequent diabetes intensification therapy. These metabolic effects were most pronounced during the first 6 months after diagnosis and diminished over time. Importantly, these metabolic disadvantages were accompanied by a significant reduction in the long-term MACE risk, with statin therapy emerging as an independent protective factor in adjusted analyses.

Our results are consistent with those of previous studies demonstrating that statin therapy modestly impairs glycemic control in patients with T2D. A meta-analysis of 9 randomized trials involving 9, 696 patients with T2D revealed that the mean HbA1c level was 0.12% higher in participants randomized to receive statin therapy than in those assigned to receive the placebo (23). A meta-analysis of data from a large sample of individual participants from the Cholesterol Treatment Trialists’ (CTT) Collaboration, which included 23 randomized trials involving more than 150, 000 participants, confirmed that statin therapy causes a dose-dependent increase in the risk of new-onset diabetes and exacerbates glycemic control in patients with T2D. Importantly, this analysis also demonstrated that the overall cardiovascular benefits of statin therapy fully outweigh any theoretical adverse effects that might arise from these small increases in glycemia (24). Notably, our study showed a greater between-group difference in glycemic improvement (0.57%) at 6 months than that reported in these previous meta-analyses. This discrepancy is likely attributable to our focus on patients with newly diagnosed T2D, who typically have better glycemic control at baseline and a greater potential for improvement with lifestyle changes and initial pharmacological therapy. Our finding that statin therapy increases the risk of diabetes therapy intensification is consistent with the literature. A large national observational study conducted in the United States revealed that compared with nonusers, statin users exhibited significantly higher rates of diabetes progression; specifically, they experienced a 41% increase in the number of antihyperglycemic medication classes used and a 16% increase in new insulin initiation (20).

From a clinical practice perspective, these findings underscore the importance of a balanced and individualized approach to statin therapy in patients with newly diagnosed T2D. Although the cardiovascular benefit of statins strongly supports their early use in most adults with diabetes, clinicians should be particularly vigilant regarding glycemic monitoring during the first 6 months after initiation, when the diabetogenic effect appeared most pronounced in our cohort. The evidence from large trials and meta-analyses suggests that statin-associated dysglycemia is not uniform across individuals. Patients with a higher baseline BMI/central adiposity, impaired fasting glucose levels (or other markers of prediabetes), and a family history of diabetes may be more susceptible to a deterioration of glycemic control after statin exposure (24–26). In such high-risk individuals, selecting statins with relatively more neutral glycemic profiles (e.g., pravastatin and possibly pitavastatin) may be considered when clinically appropriate, provided that the LDL-C goals can still be achieved (27). Moreover, the early use of glucose-lowering therapies with proven cardiovascular benefits—particularly sodium–glucose cotransporter 2 (SGLT2) inhibitors and glucagon-like peptide-1 receptor agonists (GLP-1 RAs)—represents a rational strategy to offset the metabolic risk while further strengthening cardiovascular protection (28, 29). Contemporary consensus recommendations and guidelines increasingly endorse the use of these agents in patients with established ASCVD, heart failure, CKD, or high cardiovascular risk, irrespective of the baseline HbA1c levels (30). Taken together, an approach combining early statin therapy for LDL-C reduction with early cardioprotective glucose-lowering agents may help optimize both metabolic and cardiovascular outcomes in patients with new-onset T2D.

Multiple biological mechanisms have been proposed to underlie the detrimental impact of statin therapy on glycemic homeostasis. First, statins may downregulate the expression of glucose transporter 4 (GLUT4), a key membrane protein responsible for glucose uptake in adipocytes (31, 32). Second, certain statins affect insulin secretion through direct, indirect or combined effects on calcium channels in pancreatic β-cells (33). Third, statin therapy reduces the levels of other critical downstream products, including farnesyl pyrophosphate, geranylgeranyl pyrophosphate, coenzyme Q10, and dolichol; insufficient levels of these factors impair intracellular signal transduction (34). Furthermore, statin exposure is closely linked to the development and exacerbation of peripheral insulin resistance (35). In addition, the association tends to be stronger among patients with T2D than among those with type 1 diabetes (23). Notably, the diabetogenic effect of statins is intensity dependent: compared with low-potency statins, high-intensity agents (such as rosuvastatin and atorvastatin) carry a higher risk of new-onset diabetes. Nevertheless, high-potency statins also confer greater cardiovascular protective effects, and the overall net clinical benefit remains favorable even with intensive statin regimens across most clinical populations (20, 36). Accordingly, statins remain the first-line therapeutic strategy for cardiovascular disease prevention.

This study has several limitations. First, as a retrospective observational study, it is subject to confounding by indications; patients who were prescribed statins inherently had higher baseline levels of cardiovascular risk markers (e.g., higher SBP and LDL-C levels). Although the multivariable Cox regression model was adjusted for these factors, residual confounding could not be entirely excluded. Second, new-onset T2D was defined solely based on in-hospital electronic medical records, which introduced the potential for misclassification bias. Patients with delayed diagnoses, previously undiagnosed diabetes, or those diagnosed at unaffiliated external facilities may have been erroneously categorized as incident cases. Furthermore, uniform baseline assessments, such as fundus photography or other markers of chronic diabetic damage, were not available to definitively validate the true disease duration. Third, we excluded patients who initiated statins after the baseline visit to evaluate the specific impact of an early initiation strategy. Consequently, statin exposure was not handled as a time-dependent variable, which limited our ability to assess the dynamic, long-term fluctuations in statin use and potential time-varying confounding effects. Fourth, data on specific statin types, dosages, and long-term medication adherence were not fully captured, limiting our ability to assess dose-dependent effects. Finally, this study was conducted at a single center, which may limit the generalizability of the findings to broader populations.

## Conclusions

In patients with newly diagnosed T2D, the early initiation of statin therapy is associated with significantly attenuated short-term glycemic improvement and increases the subsequent requirement for intensive glucose-lowering therapies and insulin. However, this transient metabolic disadvantage diminishes over time and is profoundly outweighed by a robust and substantial reduction in long-term major adverse cardiovascular events. These findings support the mandatory early prescription of statins to patients with T2D, coupled with vigilant early glucose monitoring and proactive adjustments to antihyperglycemic regimens to optimize both metabolic and cardiovascular outcomes.

## Data Availability

The raw data supporting the conclusions of this article will be made available by the authors, without undue reservation.
